# Narrative medicine approach for assessing the emotional experience of children and adolescents treated with daily rhGH and their caregivers

**DOI:** 10.3389/fendo.2026.1654794

**Published:** 2026-03-03

**Authors:** Domenico Corica, Cecilia Lugarà, Valentina La Malfa, Maria Pecoraro, Letteria Anna Morabito, Giorgia Pepe, Angela Alibrandi, Tommaso Aversa, Malgorzata Gabriela Wasniewska

**Affiliations:** 1 Department of Human Pathology of Adulthood and Childhood “G. Barresi”, University Hospital “G. Martino”, Pediatric Unit, University of Messina, Via Consolare Valeria 1, Messina, Italy; 2 Department of Economics, University of Messina, Messina, Italy

**Keywords:** childhood, chronic treatment, emotional burden, emotional experiences, growth hormone, narrative medicine, quality of life, therapy-related stress

## Abstract

**Introduction:**

Recombinant human growth hormone (rhGH) therapy is a long-term, injective treatment, which can be burdened by emotional burden in patients and their caregivers. Narrative medicine can help the medical team to identify patients who are experiencing this negative emotional burden. Aims of this study were: 1. To investigate the emotional experience and the Health-Related Quality of life (HRQoL) of children and adolescents treated with daily rhGH and their caregivers through a narrative medicine approach. 2. To identify the factors influencing the emotional experience.

**Methods:**

Patients on rhGH therapy for at least one year were included. Two trained psychologists asked each of the patients and their caregivers three open-ended questions. Data on emotional experience, acceptance of therapy, satisfaction and awareness of treatment, adherence, discomfort towards peers, therapy-related stress, future plans were extrapolated. Patient and parental HRQoL were also assessed.

**Results:**

Fifty-three pediatric patients were recruited (mean age 12.3 ± 2.87 years; 62.3% male; 30.2% prepubertal). Distress/anxiety was reported in 41.5% of cases. Discomfort toward peers and therapy-related stress were reported in 24.5% and 49.1% of cases, respectively, and both correlated negatively with patients’ emotional experience (p=0.019 and p=0.000, respectively) and acceptance of therapy (p=0.015 and p=0.001, respectively). Patient’s emotional experience was influenced by stress (OR 0.02, 95%CI 0.004-0.132, p= 0.000) and patient’s acceptance of therapy (OR 10.7, 95%CI 3.13-36.5, p=0.000). These latter results were confirmed by multivariate analysis (stress: OR 0.028, 95%CI 0.003-0.251, p=0.001; patient acceptance of therapy: OR 13.1, 95%CI 2.25-76.88, p=0.004, respectively, independently of age and sex.

**Conclusion:**

The emotional experience of patients on daily rhGH is negatively affected by non-acceptance of therapy and therapy-related stress. The narrative medicine approach is useful in early identification of emotional burden and promote implementation of strategies to improve psycho-social health for both patients and caregivers.

## 1 Introduction

Daily therapy with recombinant human growth hormone (rhGH) has been used for more than 40 years and has proven to be a safe and effective treatment in terms of height gain (1–4). However, this treatment is associated with several issues, including daily subcutaneous administrations and long duration of therapy. In addition, the benefits of the treatment in terms of height gain are not immediately perceived by the patient and caregivers and are strictly dependent on optimal adherence (5, 6). Despite these concerns, adherence to daily rhGH therapy has high rates in pediatric patients (7), but these patients may experience several psychological burdens, including discomfort toward their peers (8). This is closely associated to the therapy related stress and may negatively impact the quality of life (QoL) of patients and their caregivers (8–12).

In the context of personalized medicine tailored to the needs of the pediatric patient and caregivers, a promising perspective of care, is narrative medicine approach to assess the emotional distress associated with chronic treatment. Narrative medicine is a methodology applied in clinical setting that focuses on specific communication skills, where storytelling is the essential tool for examining, identifying, comprehending, and integrating the various viewpoints of all individuals involved in the experience of illness and the care process (13). In this way, it can be regarded as an important means of exploring emotional aspect. Indeed, narrative medicine can serve as valuable support for the healthcare team by helping recognize, through storytelling, the needs and emotional aspects of patients and caregivers that may not be clearly verbalized during routine check-ups (14, 15). Definitely, it is useful to identify both the strengths and weaknesses of the care experience to develop new supportive strategies aimed at improving the overall effectiveness of care, strengthening the therapeutic relationship, and minimizing anxiety and misunderstanding between the healthcare team, patients and caregivers (16). Early identification of psychosocial problems should be aimed at providing the child and caregivers with appropriate support tools to improve the overall care experience, maximize the therapeutic alliance with the care team and, consequently, treatment adherence and overall QoL (12, 17).

The aims of this study were: 1. to investigate the emotional experience and the Health-Related Quality of life (HRQoL) of children and adolescents treated with daily rhGH and their caregivers through a narrative medicine approach. 2. To identify the factors influencing the emotional experience of patients and caregivers.

## 2 Materials and methods

An observational, single-center study was carried out at the Pediatric Endocrinology Centre of the University Hospital “G. Martino” of Messina (Italy) from January to November 2024. Patients diagnosed with isolated GH deficiency (GHD) or on rhGH therapy because small for gestational age (SGA), who are treated with daily rhGH therapy for at least one year, were included. Recruitment occurred at the six-monthly follow-up visit. Inclusion criteria were: diagnosis of GHD or SGA; treatment with daily rhGH for at least one year; age over 8 years. Exclusion criteria were: genetic syndromes and/or other chronic diseases; other hormone deficiencies; neurocognitive deficits; language barrier; other chronic therapies; failure to attend routine checkups.

An interview was carried out prior to the recruitment visit to explain the objectives of the study at the parents/caregivers, and the informed consent of the parents/caregivers and the child’s assent were acquired. During the recruitment visit, past medical history and clinical data are collected, including the diagnosis and period of treatment with daily rhGH (months), the dosage of rhGH (mg/kg/day), and the number of missed daily rhGH administrations during the preceding six months. In accordance with standard treatment protocols, auxological measurements (height, weight, body mass index (BMI), height velocity, pubertal stage) and the results of biochemical tests (e.g. IGF-1) were obtained at the follow-up visit. The height was measured using a Harpenden stadiometer. The weight was recorded with a mechanical column scale equipped with movable counterweights. The BMI value was calculated using the following formula: weight (kg)/height (m2). Adherence was defined as good (one missed dose per week; >86% of doses administered) or as moderate/poor (greater than one missed dose per week; <86% of doses administered) (18).

The patients had previously undergone intellective quotient (IQ) assessment using Raven’s matrices by two specialized psychologists. At recruitment, the psychologists assessed household characteristics (whether parents were together or separated, who accompanied the child to the visit, caregivers’ education level, and presence of a caregiver at home). They also evaluated the HRQoL of patients and caregivers. Patients’ HRQoL was assessed using the KIDSCREEN-27 questionnaire, which is structured in the following five dimensions: physical well-being, psychological well-being, autonomy and parental relations, peers and social support, and school environment. Caregivers’ general HRQoL was measured using the KIDSCREEN-10 questionnaire parent version. According to the normative assessments of the KIDSCREEN manual relating to the evaluation of questionnaires, the T scores obtained were then corrected according to age and gender and interpreted according to specific reference tables (19).

Subsequently, at the same assessment, each patient and his and/or her caregiver were asked to answer three open-ended questions based on narrative medicine approach, followed by an in-depth interview.

The open-ended questions were constructed based on the following items to be investigated: emotional experience, acceptance of medical therapy, satisfaction and awareness of the treatment used, adherence, feeling of discomfort towards peers and therapy-related stress, presence and possible nature of future plans. The methodology of data collection and interpretation was based on the Grounded Theory model (20), according to the approach of Strauss and Corbin, which allows the identification of categories of interest, on which the formulation of specific questions is based, from whose answers the data of interest are obtained (21). Two psychologists carried out qualitative and quantitative analyses of individual texts through manual coding, working independently and blindly. For this analysis, they used dichotomous or three-point Likert scales, which were constructed prior to analyzing the texts in order to evaluate the predefined items (22). Specifically, a dichotomous Likert scale was employed to assess emotional experiences of the patients and the caregiver, focusing on the following aspects: well-being, signs of distress, social anxiety, and feelings of shame. In cases of disagreement or uncertainty between the two psychologists, a third independent reviewed the text to resolve discrepancies.

Finally, the quality of the responses was assessed overall in terms of the subject’s narrative competence (23). This was done by evaluating the length of the text, the vocabulary used, the coherence of the answers to the questions and the structure of the narrative using a three-point Likert scale (22).

The study cohort was stratified into subgroups to identify any potential differences between them. Patients were stratified into the following subgroups: pubertal and prepubertal, based on the assessment of pubertal Tanner stage; GHD and SGA, based on the diagnosis established before starting rhGH treatment; and finally, patients younger than 12 years and those older than 12 years of age, in accordance with Erikson’s theory of psychosocial development, which identifies age 12 as a critical age in adolescent development, characterized by the exploration of identity, the formation of self-concept and the search for one’s societal role (24).

Finally, relationships were examined between the emotional experience and therapy- related stress of the patients and the patients’ and parents’ HRQoL evaluated using the KIDSCREEN questionnaire.

The study was conducted according to Good Clinical Practice and in compliance with the Declaration of Helsinki with successive amendments. The study protocol was approved by the local Ethics Committee on January 17, 2022 (protocol number: 143 - 21).

### 2.1 Statistical analysis

Numerical variables were expressed as mean and standard deviation (SD), categorical variables as absolute frequencies and percentage. According to the Kolmogorov-Smirnov test, all variables were normally distributed. The Pearson or Spearman correlation test were performed to evaluate possible correlation among numerical or ordinal and binary variable, respectively the patient’s emotional experience, the caregivers’ emotional experience, discomfort toward peers, therapy-related stress, the patient’s acceptance of medical therapy, the caregivers’ acceptance of medical therapy, patient’s future plans, caregivers’ future plans, awareness of the treatment used, patient and the caregivers’ satisfaction with therapy, the caregivers’ therapy perception, global HRQoL of parents and global and domain-specific HRQoL of patients.

In order to evaluate potential differences in the answers between diagnosis, age class, puberal status, Student t test was applied with reference to numerical variables and Chi square test for categorical variables. Univariate and multivariate logistic regression models were estimated to identify significant predictors of emotional experience of the patient and his caregivers. The tested covariates were: age, gender, pubertal stage, duration of therapy, height gain, Raven’s Matrices, therapy-related stress, patient’s acceptance of medical therapy, caregivers’ acceptance of medical therapy, therapy adherence, patient’s awareness of therapy, patients’ future plans, caregivers’ future plans, global HRQoL of parents and global and domain-specific HRQoL of patients. We also estimated univariate logistic regression models between therapy-related stress and global HRQoL of parents and global and domain-specific HRQoL of patients. The results were expressed as Odds Ratio (OR), 95% Confidence Interval (C.I.) and p value. For all statistical analyses, a p value ≤ 0.05 was considered statistically significant. Statistical analyses were performed using IBM SPSS for Windows, Version 22 (Armonk, NY, IBM Corp.).

## 3 Results

### 3.1 Features of the population

Fifty-three patients and their caregivers were enrolled, including 62.3% males and 37.7% females. **Table 1** shows the characteristics of the population. The mean age at recruitment was 12.3 ± 2.87 years, with 66% of the patients being 12 years or older and 34% younger than 12 years. Of these, 69.8% were pubertal and 30.2% prepubertal Tanner stage. Among the patients, 69.8% received daily rhGH for isolated GHD, while 30.2% were treated for SGA. The median IQ at evaluation was 97.1 ± 13.33 points. Treatment duration averaged 65.28 ± 40.43 months, with a height gain 1.27 ± 0.83 SDS since the start of therapy. All patients were accompanied to the follow-up visit by at least one parent: 60.4% by the mother, 5.7% by the father and 34% by both parents. Parents’ education levels were 26.4% primary school, 54.7% secondary school and 17% university degree. In 45.3% of the families, at least one caregiver stayed at home, whereas 54.7% had both parents employed. Households were complete in 86.8% of cases and single parent in 13.2%.

**Table 1:** Patient and household features at the recruitment visit.

Variables	Values
**N. of patients**	53 (100%)
**Age** (years)	12.3 ± 2.87
**Dichotomic age:** < 12 y	18 (34%)
≥12 y	35 (66%)
**Gender:** boys	33 (62.3%)
girls	20 (37.7%)
**Puberal Stage:** prepubertal	16 (30.2%)
pubertal	37 (69.8%)
**Diagnosis**: GHD	37 (69.8%)
SGA	16 (30.2%)
**IQ** (points)	97.1 ± 13.33
**Therapy duration** (months)	65.28 ± 40.43
**Adherence**: good	43 (81.1%)
moderate/poor	10 (18.9%)
**Parent present during the check**: mother	33 (60.4%)
father	3 (5.7%)
both parents	18 (34%)
**Parent’s level of education**: middle school	14 (26.4%)
high school	29 (54.7%)
university degree	9 (17%)
**Home parent**: yes	24 (45.3%)
no	29 (54.7%)
**Household**: complete	46 (86.8%)
incomplete	7 (13.2%)

Data are reported in frequency and percentage or mean value ± SDS.

Bold values denote the primary variables included in the analysis, whereas non-bold values indicate their corresponding categories.

Intellective quotients (IQ); growth hormone deficiency (GHD); small for gestational age (SGA).

### 3.2 Adherence and HRQoL

At the time of the medical interview, good treatment adherence was reported in 81.1% of cases, while in 18.9% of cases, patient and/or caregiver reported that <86% of the prescribed daily rhGH doses had been administered in the previous six months (moderate/poor adherence).

Assessment of patients’ HRQoL using the KIDSCREEN-27 revealed the following scores across five dimensions: physical well-being 54.66 ± 10.17; psychological well-being 52.45 ± 8.58; autonomy and parental relationships 52.62 ± 8,61; peer and social support 52.26 ± 11.86; school environment 52.15 ± 8.27. Patients’ general HRQoL was 51.49 ± 8.32. Parents’ general HRQoL assessed by the KIDSCREEN- 10 parent version was 52.83 ± 7.26.

Based above-mentioned results, 84.3% of patients reported a good global HRQoL, instead 13.7% exhibited a partially positive HRQoL; no patients reported low HRQoL. Regarding caregivers, 77.4% had a good overall HRQoL, 17% a partially positive HRQoL and 5.7% reported a low HRQoL.

### 3.3 The narrative of the care experience: analysis of patients’ and caregivers’ contributions

The qualitative analysis of the narrative texts obtained from the answers to the open-ended questions indicated that 24.5% of responses were of high quality, 49.1% were of good quality and 26.4% were of low quality.

Concerning the assessment of the patients’ emotional experience, it was found that 41.5% of the patients experienced discomfort, stress, anxiety or shame during treatment, instead 58.5% reported positive emotional experience (see **Table 2**). Additionally, 24.5% expressed discomfort towards peers due to ongoing chronic treatment and in 49.1% a condition of therapy-related stress was reported. With regard to the assessment of the caregivers’ emotional experience, 32.1% report feelings of discomfort, instead 67.9% declared positive emotional experience in relation to their child’s therapy. Patient’s acceptance of treatment was rated as optimal in 56.6% of cases, moderate in 30.2% and poor in 13.2%. Among caregivers, acceptance of the treatment was rated optimal in 83% of cases and poor in 17% (no caregiver reported moderate treatment acceptance).

**Table 2:** Selected narrative extracts from pediatric patients undergoing rhGH therapy and their caregivers.

Patients’ stories	
Positive emotional experience (58.5%)	Negative emotional experience (41.5%)
*“I’m normal, I don’t feel uncomfortable in therapy. I don’t have a problem with it. I remind my mother of it. I do it to grow.”*	*“The feeling is obviously that of a “ball and chain”, because obviously the needle restricts me in so many ways.”*
*“Having the injections every day is now normal for me. I don’t feel uncomfortable at all and I’m happy with the results.”*	*“I only feel uncomfortable when we are somewhere and I have to do it “in secret”.”*
*“I’ve got used to this growth hormone therapy now and I have to be honest, it doesn’t make me uncomfortable. I’m quietly going through this therapy.”*	*“I get tired every day. When I go on holiday with my parents, I’m tired and I don’t want to go to school. I don’t want to do it with other people in the room.”*
Well-being in peer relations (75.5%)	Discomfort towards peers (24.5%)
*“I also get on well with my friends and lead a very normal life.”*	*“Yes, sometimes I think I am different from others. Because maybe, I can be teased”*
*“I don’t think this makes me different from all my peers.”*	*“Yes, therapy makes me uncomfortable and that’s why I don’t talk about it with others (apart from my family).”*
*“My classmates have motivated me from the beginning and it’s also thanks to them that it doesn’t cause me discomfort to take shots every day.”*	*“Yes, I feel short; it is having little effect in my opinion, I would like to stand up even a few centimeters.”*
Acceptance of the treatment (86.8%)	Non-acceptance of the treatment (13.2%)
*“I am aware of doing this treatment because it is something that helps me to grow and I am very motivated to do the injections regularly.”*	*“Sometimes I am motivated. Other times I am not. I feel uneasy even if I have to do it.”*
*“I’m used to wearing/doing the sting by now, so it doesn’t bother me. I like it because it helps me to grow.”*	*“I don’t like doing the injection. I’m bored with doing the injection.”*
*“I have very positive feelings about this treatment because it has helped me a lot to feel better especially with myself.”*	*“I feel uncomfortable.”*

Caregivers’ stories	
Positive emotional experience (67.9%)	Negative emotional experience (32.1%)
*“Overall, we are satisfied and grateful that we still have the opportunity to care for our son and hope that everything turns out for the best”*	*“It is certainly not a nice experience, but we are aware of the benefits our daughter gets, and we are motivated to continue it. It is often a source of discomfort for both of us as parents and our daughter.”*
*“Very positively and we saw many advantages.”*	*“My son doesn’t accept the administration willingly; he experiences it as a frustrating experience despite being aware of the necessity. I hope it isn’t necessary to continue beyond the growth period.”*
*“It was a “good experience” for me because I got some great results.”*	*“At first it was heavier, after six years of therapy we no longer pay attention to it, although it is sometimes an inconvenience, especially when we go on holiday.”*

About satisfaction with the treatment outcomes, 50.9% of the patients reported being satisfied, 3.8% expressed dissatisfaction and 45.3% did not comment. In almost all cases (96.2%), the caregivers reported satisfaction with the results of the rhGH treatment.

Awareness of the importance adhering to therapy emerges in 75.5% of the patients’ answers and in 100% of the caregivers’ answers. Moreover, 88.7% of the caregivers acknowledged the need for good adherence to achieve maximum benefit from therapy, while 11.3% lacked this awareness and neglected the consequences of poor adherence. As for future plans, 69.8% of patients expressed clear vision, instead 30.2% of them were uncertain. Among caregivers, 83% foresaw a future perspective of well-being for their child, instead the 17% reported a perspective of uncertainty and feeling uneasy.

### 3.4 Correlation analysis

The results of the correlation analysis are reported in **Table 3**. The patient’s positive emotional experience was negatively correlated with both discomfort toward peers in relation to chronic therapy (r= -0.3207; p= 0.019) and therapy-related stress (r= -0.4472; p= 0.000). Patients’ positive emotional experience positively correlates with positive emotional experience reported by caregivers (r= 0.4055; p=0.003) and with patients’ acceptance of therapy (r=0.6481; p=0.000). Caregivers positive emotional experience shows a positive correlation with patient’s acceptance of therapy (r= 0.3421; p=0.012) and a negative correlation with therapy-related stress (r= -0.2960; p=0.031). Discomfort toward peers is directly correlated to therapy-related stress (r=0.3178; p=0.020) and negatively related to acceptance of therapy (r= -0.3340; p=0.014). Therapy-related stress also correlates with reduced patient acceptance of therapy (r= -0.4472; p= 0.001). No correlations were found between the patient’s or caregivers’ emotional experience and the patient’s global HRQoL, nor in the scores of the five dimensions of the KIDSCREEN - 27, nor with the parent’s global HRQoL.

**Table 3:** Correlation analysis.

Patient acceptance of therapy	1											
Patients’ emotional experience	*r* 0.6481 *p* 0.0000	1										
Therapy- related stress	*r* -0.4472 *p* 0.0008	*r* -0.7053 *p* 0.0000	1									
Discomfort toward peers	*r* -0.3340 *p* 0.0145	*r* -0.3207 *p* 0.0192	*r* 0.3178 *p* 0.0204	1								
Patient awareness	r 0.0371. p 0.7919	r-0.1243 p 0.3753	r 0.0331 p 0.8140	r 0.0192 p 0.8913	1							
Patients’ future prospective	r-0.0696 p 0.6206	r0.1133. p 0.4192	r-0.1768 p 0.2053	r-0.1027 p 0.4642	r 0.1027 p 0.4642	1						
Patient’s satisfaction therapy	r0.1868 p0.3319	r0.0677 p0.7271	r-0.0283 p 0.8841	r-0.2360 p 0.2177		r-0.1535 p 0.4266	1					
Caregiver emotional experience	*r* 0.3421 *p* 0.0122	*r* 0.4055 *p* 0.0026	*r* -0.2960 *p* 0.0314	r -0.1719 p 0.2183	r -0.1099 p 0.4334	r -0.0116 p 0.9341	r 0.0677 p 0.7271	1				
Caregiver acceptance of therapy	r 0.3402 p 0.0127	r 0.0269 p 0.8481	r -0.0588 p 0.6758	r -0.0926 p 0.5098	r -0.0242 p 0.8632	r -0.0785 p 0.5764	r 0.2858 p 0.1329	r 0.2275 p 0.1013	1			
Caregiver future prospective	r 0.0425 p 0.7624	r 0.1289 p 0.3575	r -0.1593 p 0.2545	r -0.0926 p 0.5098	r -0.0242 p 0.8632	r-0.0785 p 0.5764	r-0.1390 p 0.4720	R 0.1198 p 0.3927	r-0.0707 p 0.6149	1		
Caregiver satisfaction therapy	r 0.0838 p 0.5508	r 0.2351 p 0.0902	r -0.2018 p 0.1473	*r* -0.3474 *p* 0.0108	r 0.1172 p 0.4031	r 0.0855 p 0.5429	r-0.0514 p 0.7910	r-0.1361 p 0.3312	r-0.0896 p 0.5236	*r* 0.4379 *p* 0.0010	1	
Caregiver awareness	*r* 0.3024 *p* 0.0278	r 0.0616 p 0.6614	r 0.1124 p 0.4230	r -0.0731 p 0.6028	r 0.0731 p 0.6028	r 0.1542 p 0.2703	r 0.3544 p 0.0593	r 0.0096 p 0.9454	*r* 0.6314 *p* 0.0000	r -0.1616 p 0.2477	r -0.0708 p 0.6146	1
	Patient acceptance of therapy	Patients emotional experience	Therapy‐related stress	Discomfort toward peers	Patient awareness	Patients future prospective	Patient satisfaction therapy	Caregiver emotional experience	Caregiver acceptance of therapy	Caregiver future prospective	Caregiver satisfaction therapy	Caregiver awareness

Color shading: blue cells denote non-significant correlations (p > 0.05); green cells denote statistically significant correlations (p ≤ 0.05); red cells indicate diagonal elements representing the correlation of each variable with itself.

### 3.5 Regression models

No statistically significant linear association was found between patients’ emotional experience or therapy-related stress as predictors and patients’ or caregivers’ HRQoL, including the global score and all five domain scores of the KIDSCREEN – 27 questionnaire.

The univariate logistic regression analysis, considering the patients’ emotional experience as the dependent variable, shows a significant association with therapy-related stress (OR 0.024, 95%CI 0.004 - 0.132; p=0.000) and with patients’ acceptance of therapy (OR 10.700, 95%CI 3.135 - 36.518; p= 0.000). These associations are supported by multivariate logistic regression model, which confirms the association between patients’ emotional experience and therapy-related stress (OR 0.028 and 95% CI 0.003 - 0.251; p= 0.001) as well as with patients’ acceptance of therapy (OR 13.161 and 95% CI 2.253 - 76.883; p= 0.004) (**Table 4**).

**Table 4:** Univariate and multivariate regression analysis for patients’ emotional experience.

Variables	Association measures	Univariate regression	Multivariate regression*
Chronic therapy-related stress	OR	0.024	0.028
	95% IC	0.004 – 0.132	0.003 – 0.251
	*p value*	0.000	0.001
Patients’ acceptance of therapy	OR	10.700	13.161
	95% IC	3.135-36.518	2.253 – 76.883
	*p value*	0. 000	0.004

*The multivariate regression model included the following variables: age, gender, chronic therapy related stress, patient acceptance therapy.

### 3.6 Subgroup comparison analysis

A comparison between patients younger than 12 years and those older than 12 years indicates that the latter had significantly higher distress towards peers associated with chronic therapy (p = 0.016).

There were no statistically significant differences between GHD and SGA patients and between pubertal and prepubertal patients (data not showed).

## 4 Discussion

The present study documented that the emotional experience of patients on daily rhGH is negatively affected by non-acceptance of therapy and therapy-related stress.

Therefore, although the patients in this study had both good adherence and good HRQoL across the five different dimensions of KIDSCREEN27, through a narrative medicine approach a significant percentage of patients (41.5%) and caregivers (32.1%) who had a negative emotional experience, characterized by distress and anxiety, with respect to chronic drug therapy emerged and therapy related stress.

Maghnie et al. documented low scores in the social and emotional items in the HRQoL assessment of adolescents with GHD and their caregivers, although the overall score attested to good HRQoL (11). In this context, a narrative medicine approach can be critical in bringing out important aspects related to the emotional experience of the chronically ill patient that would not otherwise emerge in routine medical checkups.

A narrative medicine approach was previously applied in the Italian CRESCERE project study to explore the emotional and psychological experiences of children with GHD undergoing therapy, their caregivers, and the healthcare professionals supporting them. The narratives showed the presence of discomfort and intolerance toward chronic therapy, especially by adolescents, despite a good degree of awareness about the importance of treatment. The authors highlighted the importance of a personalized therapeutic approach tailored to the patient and caregivers, of effective communication, and of the necessity for constant educational and emotional support for families (25).

Therapy-related stress was reported in a significant percentage of patients in our study (49.1%) and was found to be among the factors that mainly influenced emotional experience of patients, independently of gender and diagnosis (GHD or SGA).

A meta-analysis by Pinquart and Shen reveals that children and adolescents with chronic illnesses exhibit significantly higher levels of psychosocial problems compared to their healthy peers, particularly concerning anxiety, depression, aggressiveness, and behavioral disorders (26). In addition to the emotional burden associated with any chronic condition, the requirement for daily treatment can induce an emotional experience characterized by discomfort and stress in pediatric patients (8). This negative experience often involves and affects the entire household (17, 27, 28).

In our study, another factor that was significantly related to emotional experience of patients was discomfort toward peers in relation to chronic therapy, particularly in patients aged 12 years or older. Adolescence is a period during which the risk of emotional vulnerability is higher than in childhood, especially among subjects undergoing chronic therapies. Adolescents with chronic conditions often experience increased social anxiety, and shame related to treatment (9, 29). Recently, Aryayev et al, aiming to identify psychological problems in children with GHD by the Goodman’s Strengths and Difficulties Questionnaire, demonstrated that these children more frequently exhibit abnormal scores in the “total difficulties”, “emotional problems” and “peer problems” scales compared to their non-GHD peers, indicating a higher risk of psychological and behavioral issues (9). Addressing this aspect in routine follow-up is crucial, as positive social relationships have been shown to act as protective factors for mental health in children with chronic illnesses (30). Furthermore, La Greca et al. emphasized that children with stigmatizing medical conditions or central nervous system involvement may face difficulties in peer relationships and highlighted the importance of social support from friends and peers which appears to facilitate adaptation to illness and helps children modify their lifestyles to observe the therapeutic regimens (31).

Acceptance of therapy by the patient and caregiver represent another key aspect in the assessment of the pediatric patient undergoing chronic therapy. In this study, acceptance of therapy represents one of the factors most influencing emotional experience of patients, being positively associated with positive emotional experience of chronic therapy. Therapy acceptance should be regarded as an adaptive coping mechanism, enabling the patient and caregivers to adjust to the chronic condition and its daily management, thus mitigating its psychological impact (32).

Lastly, it is essential to reinforce that pediatric care requirement center not only on the child but also on the caregiver (33). Our data demonstrated a correlation between the positive emotional experiences of the caregiver and the child, as well as between caregiver awareness of the importance of therapy and the child’s acceptance. Furthermore, the caregiver’s positive emotional experience was directly associated with lower therapy-related stress in the patient. These findings are supported by a recent meta-analysis assessing family functioning and medical adherence in children and adolescents with chronic health conditions, which concluded that family functioning is significantly correlated with adherence (34). Moreover, another recent systematic review with meta-analysis indicated that psychological interventions based on Acceptance and Commitment Therapy improve mental health outcomes in caregivers and reduce emotional and behavioral problems in children (35).

Patients and their caregivers should be considered central to the care process. During follow-up, it would be advisable to check periodically, with the support of a psychologist if possible, the presence of the following situations: potential difficulties the family may encounter with daily injections; problems related to the injection device; patient’s behavior and general acceptance of the treatment; fear of needles as well as possible feelings of shame; discomfort or social relationship with peers. It is important that patients, especially adolescents, feel that their point of view is valued by the healthcare team. Regarding the strategies to be adopted, the care assistance should be personalized according to individual patient needs, and each clinical encounter should promote constructive and effective dialogue aimed at early identification of psychosocial burdens. Psychological support and interventions tailored to improve emotional well-being and family support should be essential parts of the therapeutic approach to promote better treatment acceptance, positive peer relationships, and overall good HRQoL (10, 36–38).

Ultimately, it is important to consider how the availability of a long-acting formulation might affect the therapeutic experience of pediatric patients undergoing rhGH therapy. Although the current lack of real-word data on the emotional experience, therapy-related stress, and therapeutic adherence in children on long-acting growth hormone (LAGH) therapy, the results of currently available clinical trials and the recent Consensus Statement on the use of LAGH treatment in paediatric patients with GHD suggest that LAGH could offer children and their caregivers a reduction in the burdens associated with chronic treatment, enhancing treatment adherence, clinical outcomes, emotional experience, and quality of life (39–41).

The strengths of this study derive from the selection of the study population. The cohort consisted of a homogeneous group of Caucasian pediatric patients, balanced by sex and age, indeed include both children and adolescents. All participants had been receiving daily rhGH therapy for a minimum of 12 months and were in follow up at the same clinical Center. To minimize potential confounding factors related to the need for complex or multimodal therapeutic regimens, individuals with multiple pituitary hormone deficiency or other know chronic conditions were ruled out. All patients had a normal range IQ and were enrolled in standard education programs, enabling them to the ask to the question independently. The data collection process from the involvement of two experienced phycologists who provided support to both participants and their caregivers during the administration of the questionnaires, thereby improving response accuracy and participant compliance.

This study has some limitations, including a small sample size and the observational design. The lack of standardized and validated tools for extracting data from narrative medicine texts, as well as the potential for subjective interpretative bias, were mitigated through independent coding of the narrative texts by two psychologists.

## 5 Conclusions

Emotional experience of patients receiving daily rhGH therapy are negatively impacted by non-acceptance of therapy and therapy related stress. Lack of therapy acceptance emerged as a key factor linked to negative emotional experiences, therapy-related stress, and peer-related discomfort. Narrative medicine has proven to be a valuable tool in optimizing care, allowing the identification of psychosocial issues that may negatively influence treatment adherence and QoL in children and adolescents on chronic therapy, as well as their caregivers. Through this approach, the health care team should recognize early the emotional burden to implement targeted supportive strategies aimed at promoting psycho-social health for both patients and their families.

Narrative medicine should become routine practice in clinical settings for the treatment of patients with chronic diseases, with the aim of improving communication between patients, their families, and healthcare teams. Future real word studies will help validate our findings.

## Data Availability

The raw data supporting the conclusions of this article will be made available by the authors, without undue reservation.
